# Influenza virus activation of the interferon system

**DOI:** 10.1016/j.virusres.2015.02.003

**Published:** 2015-11-02

**Authors:** Marian J. Killip, Ervin Fodor, Richard E. Randall

**Affiliations:** a Biomedical Sciences Research Complex, University of St Andrews, North Haugh, St Andrews, Fife KY16 9ST, UK; b Sir William Dunn School of Pathology, University of Oxford, South Parks Road, Oxford OX1 3RE, UK

**Keywords:** Influenza viruses, Innate immunity, Interferon, RIG-I

## Abstract

The host interferon (IFN) response represents one of the first barriers that influenza viruses must surmount in order to establish an infection. Many advances have been made in recent years in understanding the interactions between influenza viruses and the interferon system. In this review, we summarise recent work regarding activation of the type I IFN response by influenza viruses, including attempts to identify the viral RNA responsible for IFN induction, the stage of the virus life cycle at which it is generated and the role of defective viruses in this process.

## Highlights

• We summarise the literature regarding activation of the IFN response by influenza viruses.
• We consider evidence concerning the identity of the viral RNA responsible for IFN induction.
• The link between IFN induction and defective virus genomes is discussed.

## 1 Introduction

The influenza viruses are a leading cause of respiratory illness in humans and are responsible for annual seasonal outbreaks that have serious economic impact. In addition, the wide host range of the virus and the potential for genome reassortment between human influenza viruses and those of other species mean these viruses present a pandemic threat. To establish a productive infection and thus cause disease, influenza viruses must overcome host innate immune responses that are very rapidly activated during infections. The interferon (IFN) family of antiviral cytokines play a major role in these responses and are critical in restricting the early stages of virus infections prior to the activation of the adaptive immune system. The IFNs and other innate immune mediators have serious implications for the outcome of influenza virus infections in terms of disease severity, since highly pathogenic viruses are often associated with excessive cytokine responses (Baskin et al., 2009, Kash et al., 2006). In this review, we summarise recent developments in our understanding of how influenza viruses activate the IFN system and highlight areas in which this understanding is still incomplete.

## 2 The interferon response to influenza virus

The IFNs are expressed and secreted following the detection of viral components within infected cells; subsequent binding of IFN to its cognate receptor at cell surfaces leads to the upregulation of hundreds of different interferon-stimulated genes (ISGs) that establish an ‘antiviral state’ to efficiently limit further replication and spread of the virus (reviewed in Randall and Goodbourn, 2008). Of these ISGs, several have been identified as having direct anti-influenza virus activity. These include the Mx family of GTPases, which are thought to form oligomeric rings around viral nucleocapsids to inhibit their nuclear import and/or replication (Gao et al., 2011, Haller et al., 1980, Pavlovic et al., 1992, Turan et al., 2004, Xiao et al., 2013, Zimmermann et al., 2011); viperin, which affects virus budding and thus limits the release of viral particles from infected cells (Wang et al., 2007) (although the contribution of viperin to the restriction of influenza virus replication in vivo is less clear-cut, Tan et al., 2012); and the IFN-induced transmembrane (IFITM) family members (in particular IFITM3), which interfere with fusion between viral and endosomal membranes thereby limiting viral entry (Brass et al., 2009, Desai et al., 2014, Everitt et al., 2012, Feeley et al., 2011, Li et al., 2013).

The IFNs are classified into three types according to their amino acid sequence and the type of receptor through which they signal. The type I IFNs (which include multiple IFN-α subtypes and IFN-β) and the type III IFNs (IFN-λ) are directly upregulated following virus infection; these are the major IFNs secreted following influenza virus infections in vitro and in vivo (Crotta et al., 2013, Ioannidis et al., 2013, Jewell et al., 2010, Khaitov et al., 2009, Wang et al., 2009). In contrast, type II IFN (IFN-γ) is secreted by activated T lymphocytes and NK cells and will not be discussed further in this review. Airway epithelial cells, plasmacytoid dendritic cells (pDCs) and macrophages are the main producers of IFN during influenza virus infections (Cheung et al., 2002, Hogner et al., 2013, Ioannidis et al., 2013, Jewell et al., 2007, Kallfass et al., 2013, Kaminski et al., 2012). Several studies suggest that airway epithelial cells are primarily responsible for type III IFN production while dendritic cells and alveolar macrophages are the major source of type I IFNs (Ioannidis et al., 2013, Khaitov et al., 2009, Wang et al., 2009). The induction of both type I and type III IFNs in virus-infected epithelial cells is thought to occur in response to the same viral ligands and via the same signalling components (Crotta et al., 2013, Onoguchi et al., 2007); furthermore, the panel of ISGs upregulated in airway epithelia in response to type I and type III IFNs following influenza virus infection shows complete overlap (Crotta et al., 2013). However, while the type I IFN receptor is expressed at the surface of all cells, expression of the type III IFN receptor is primarily restricted to epithelial cells in the respiratory and gastrointestinal tracts (Mordstein et al., 2010, Sommereyns et al., 2008). During an influenza virus infection, it is possible that this differential expression of the type III IFN receptor functions to elicit an antiviral response in airway epithelia while restricting the activation of immune cells and associated lung pathology (Hogner et al., 2013).

The IFN induction cascade becomes stimulated following the recognition of certain molecular structures that are absent in uninfected cells, termed pathogen-associated molecular patterns (PAMPs). For RNA virus infections, these PAMPs are predominantly certain features of viral RNA that are not usually present in cellular RNAs, such as regions of double-stranded RNA (dsRNA) or the presence of a 5′-triphosphate (5′ppp) or a 5′-diphosphate (5′pp) group. The pathogen-recognition receptors (PRRs) play a critical role in the distinction of self from non-self molecules by binding to PAMPs within infected cells. Two types of PRRs have been implicated in the induction of IFNs by influenza viruses, with the role of a particular PRR depending on the cell type and nature of the viral stimuli. The Toll-like receptor (TLR) family of transmembrane proteins are able to recognise a wide range of microbial ligands, and signal downstream through either MyD88 (myeloid differentiation primary response 88) or TRIF (Toll-interleukin-1 receptor (TIR)-domain-containing adaptor inducing interferon (IFN)-β) adaptor proteins to stimulate IFN expression, depending on the particular TLR (reviewed in Lester and Li, 2014). TLR7 is a receptor for ssRNA and recognises incoming virions in endosomal compartments during influenza virus infection of pDCs (Diebold et al., 2004, Lund et al., 2004); it is consequently required for the production of high levels of type I IFNs by these cells (Diebold et al., 2004, Kaminski et al., 2012, Lund et al., 2004). In other cell types, TLR7 is dispensable for IFN induction by RNA viruses including influenza virus (Kato et al., 2005, Koyama et al., 2007, Yoneyama et al., 2004), either because it is not expressed (Mayer et al., 2007) or because its localisation is non-endosomal (Ioannidis et al., 2013). TLR3 recognises dsRNA and is also activated by influenza virus infection (Le Goffic et al., 2007). However, although TLR3 contributes to IFN induction by other viruses (reviewed in Lester and Li, 2014), TLR3-deficient airway epithelial cells and pDCs have normal type I IFN responses to influenza virus infection (Diebold et al., 2004, Le Goffic et al., 2007); instead, TLR3 in the context of influenza virus infections functions to activate pro-inflammatory signalling pathways (Guillot et al., 2005, Le Goffic et al., 2007).

Non-self RNAs in the cytoplasm are detected by a second group of PRRs, the cytosolic RIG-I-like receptors (RLRs), consisting of RIG-I, MDA-5 and LGP2. These receptors are essential for IFN induction during RNA virus infections of non-pDC cell-types, and mice that are deficient in RLR signalling pathways are extremely susceptible to RNA viruses despite possessing intact TLR systems (Kato et al., 2006, Kumar et al., 2006, Sun et al., 2006). Both RIG-I and MDA-5 contain two caspase activation and recruitment domains (CARDs) at their N-terminus, an RNA helicase domain possessing dsRNA-dependent ATPase activity and a C-terminal regulatory domain (CTD). Ligand binding to the CTD and the helicase domain induces a conformational change in the RLRs that exposes the CARDs (Kowalinski et al., 2011, Lu et al., 2010, Luo et al., 2011, Wang et al., 2010). Activity following ligand binding is modulated by the posttranslational modification of the CARDs by dephosphorylation and/or ubiquitination (reviewed in Gack, 2014). For example, TRIM25, RIPLET and/or MEX3C-dependent Lys63-linked ubiquitination of the CARDs occurs following ligand binding to RIG-I (Gack et al., 2007, Kuniyoshi et al., 2014, Oshiumi et al., 2009, Oshiumi et al., 2010). Additionally, free Lys63 polyubiquitin chains have been reported to bind RIG-I and MDA-5 CARDs and stimulate activity (Zeng et al., 2010). Downstream signal transduction occurs by the CARD-dependent association of RIG-I/MDA-5 with the mitochondrial antiviral signalling protein (MAVS) at the outer mitochondrial membrane. This interaction promotes MAVS oligomerisation and the assembly of large multiprotein complexes that activate NF-κB and the IFN regulatory factors (IRF3 or IRF7, depending on the IFN subtype, Osterlund et al., 2007) to stimulate expression of type I and type III IFNs. LGP2 has a similar structure to RIG-I and MDA-5 but lacks the CARDs; this protein has thus been implicated in the regulation of RIG-I and MDA-5 activity (Childs et al., 2013, Saito et al., 2007, Satoh et al., 2010).

RIG-I and MDA-5 each recognise different types of viral RNA, with RIG-I most efficiently activated by short stretches of 5′ppp or 5′pp dsRNA while MDA-5 is activated by longer stretches of dsRNA in a 5′ phosphate-independent manner; these PRRs have therefore been shown to recognise different families of RNA viruses (Kato et al., 2006, Loo et al., 2008). Knockdown studies have shown that IFN induction in response to influenza virus infection is predominantly mediated through RIG-I (Kato et al., 2006, Loo et al., 2008), and influenza virus-induced IFN expression in airway epithelial cells, the primary site of influenza virus replication, is RIG-I-dependent (Le Goffic et al., 2007). MDA-5 may still contribute to IFN induction by influenza viruses however, since small reductions in IFN induction and ISG responses can be seen in infected MDA-5-deficient cells compared to control cells (Husser et al., 2011, Kato et al., 2006, Loo et al., 2008). Moreover, type I IFNs are still produced following influenza virus infection or transfections of influenza RNA in chicken cells, which lack RIG-I (Karpala et al., 2011, Liniger et al., 2012). Instead, IFN induction in this system is mediated through MDA-5 (Liniger et al., 2012).

## 3 Influenza virus replication

The influenza virus genome is composed of eight different segments of single-stranded negative-sense viral RNA (vRNA) that exists in virions in the form of viral ribonucleoproteins (vRNPs) which are the minimum units required for viral transcription and replication (Shaw and Palese, 2013). vRNPs are held in a closed circular conformation by the binding of the trimeric viral RNA-dependent RNA polymerase to the partially complementary 5′ and 3′ termini of the vRNA, with the remainder of the vRNA segment bound along its length by viral nucleoprotein (NP) oligomers (reviewed in Resa-Infante et al., 2011). Following binding of virions to sialic acid-containing receptors on the surface of host cells, virus particles are taken into the cell by endocytosis followed by vRNP release into the cytoplasm and import to the nucleus, where they serve as the templates for viral transcription and replication. vRNPs are first transcribed by the vRNP-associated polymerase in a reaction primed by 5′ capped 10–13nt RNAs that are derived from cellular pre-mRNAs through the polymerase cap-binding and endonucleolytic activities (reviewed in Fodor, 2013). Viral mRNA synthesis is therefore dependent on the activity of host RNA polymerase II and can thus be inhibited by drugs that target cellular transcription (Chan et al., 2006, Hay et al., 1977, Mark et al., 1979, Vreede et al., 2011). The viral polymerase is also responsible for polyadenylating the 3′ end of viral transcripts, following which they are exported to the cytoplasm for viral protein expression. Replication of the influenza virus genome occurs through the synthesis of full-length complementary copies of the vRNA, the complementary RNA (cRNA). cRNAs are also encapsidated into cRNPs, which in turn act as the template for the polymerase-directed synthesis of progeny vRNPs. Since genome replication is an unprimed reaction and occurs de novo, influenza virus vRNAs and cRNAs possess a 5′ppp.

## 4 Evasion of PRR recognition by influenza viruses

**Fig. 1:**
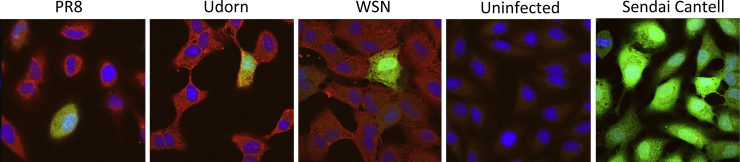
Heterogeneity in activation of the IFN induction cascade in cells infected with influenza viruses. A549 cells expressing GFP under the control of an IFN-β promoter (A549/pr(IFN-β).GFP; Chen et al., 2010) were infected with 5 PFU/cell of PR8, Udorn or WSN strains of influenza A virus. Cells were left uninfected or infected with 5 PFU/cell Sendai virus (Cantell strain) as negative and positive controls for GFP expression respectively. At 16 h post-infection, monolayers fixed and stained with antibody raised against disrupted X31 virus and DAPI; GFP (green), viral protein expression (red) and cell nuclei (blue) were subsequently examined by confocal microscopy.

If naked cRNAs and vRNAs were generated during the replication of the influenza virus genome, cRNA:vRNA hybridisation would lead to the formation of RIG-I and MDA-5 ligands, activation of the IFN response and restriction of virus replication. To avoid this, influenza virus employs strategies to evade detection by RLRs, thus allowing the virus to replicate efficiently without inducing IFN. Firstly, unlike other RNA viruses that replicate exclusively in the cytoplasm, influenza virus transcription and replication is nuclear. Since RIG-I and MDA-5 are confined to the cytoplasm, nuclear genome replication means that cRNA and progeny vRNAs are generated at a site that is inaccessible to these PRRs. Secondly, the synthesis of full-length cRNA from vRNA and vice versa is very closely coupled to encapsidation; the interaction between the first nucleoprotein monomer and polymerase at 5′ end of the RNA is thought to nucleate the sequence-independent encapsidation of the nascent RNA (Ye et al., 2006). Consequently, NP is required for full-length genome amplification (Honda et al., 1988, Mena et al., 1999, Shapiro and Krug, 1988, Vreede et al., 2011). This process also appears to involve cellular factors, since UAP56 and Tat-SF1 interact with NP to promote viral RNA encapsidation (Momose et al., 2001, Naito et al., 2007). Thus, nascent full-length cRNA and vRNA molecules do not exist as ‘free’ RNA and the formation of cRNA:vRNA hybrids is prevented. Consistent with this, long dsRNAs are undetectable during influenza virus infections (Bonin et al., 2000, Kato et al., 2008, Pichlmair et al., 2006, Weber et al., 2006) and the knockdown of UAP56 leads to an accumulation of dsRNA in infected cells (Wisskirchen et al., 2011). Thirdly, influenza virus encodes antagonists of the IFN system (reviewed in Garcia-Sastre, 2011). The principal of these is the NS1 protein, which targets multiple components of the IFN induction cascade to limit IFN expression (reviewed in Engel, 2013, Hale et al., 2008). NS1 sequesters dsRNA to limit activation of RLRs (Donelan et al., 2003, Steidle et al., 2010), targets TRIM25 to inhibit RIG-I activation (Gack et al., 2009) and in some influenza virus strains, limits IFN expression post-transcriptionally by interfering with the processing and nuclear export of cellular mRNAs (Fortes et al., 1994, Hayman et al., 2006, Nemeroff et al., 1998, Noah et al., 2003, Satterly et al., 2007). Other viral proteins have also been described as having IFN-antagonistic properties, including PB1-F2 (Dudek et al., 2011, Varga et al., 2011) and the viral polymerase (Graef et al., 2010, Iwai et al., 2010, Vreede et al., 2010). Nuclear replication, efficient encapsidation of cRNA and vRNA, and IFN antagonism by viral proteins are very effective at preventing activation of the IFN system during infection: studies examining IFN induction in individual cells infected with a range of strains of influenza A virus found that the IFN-β promoter had only been activated in a small proportion of infected cells, both in vitro and in vivo (Fig. 1) (Chen et al., 2010, Kallfass et al., 2013). Furthermore, activation of the IFN response could not be detected within some developing influenza virus plaques in lung epithelial cells (Chen et al., 2010). This may also be the case for influenza viruses that do not express a functional NS1 protein and therefore lack the principal viral IFN antagonist. Such viruses are known to be better inducers of IFN than wild-type viruses (Egorov et al., 1998, Garcia-Sastre et al., 1998, Kochs et al., 2009, Wang et al., 2000), yet Kallfass et al. (2013) demonstrated IFN-β promoter activation in only a fraction of infected cells within the lung epithelia of IFN-β reporter mice infected with a virus lacking the NS1 gene. Thus, the elevated IFN expression induced by this and other NS1-defective viruses relative to wild-type virus is likely to be due to a subset of infected cells (as has been observed for cells infected with a paramyxovirus lacking an IFN antagonist, Killip et al., 2011), and this is sufficient for the establishment of an antiviral state that impairs the replication of these viruses in IFN-competent systems (Egorov et al., 1998, Garcia-Sastre et al., 1998). The reasons for this cell-to-cell variation in IFN-β promoter activation by influenza are not yet fully resolved, but may be dictated by differences in the nature of the infecting virus particles (i.e. the presence of defective interfering [DI] viruses, which will be discussed later in this review, Baum et al., 2010, Chen et al., 2010, Killip et al., 2011, Tapia et al., 2013), the host cell (Rand et al., 2012, Zhao et al., 2012), or indeed a combination of both, depending on the cell system and the virus used.

## 5 In vitro RIG-I ligands

Several different types of RNA have been identified as ligands for RIG-I in the literature (reviewed in Schlee et al., 2009). The presence of a triphosphate at the 5′ end of RNA is now generally accepted as a particularly important characteristic in a RIG-I ligand (Hornung et al., 2006, Kowalinski et al., 2011, Lu et al., 2010, Wang et al., 2010), although it has been demonstrated recently that RIG-I can additionally trigger IFN induction in response to RNA bearing 5′-diphosphates (Goubau et al., 2014). Other features such as double-strandedness, length and A/U composition can also define the ability of RNA to induce IFN through RIG-I, irrespective of 5′ phosphates (Binder et al., 2011, Davis et al., 2012, Hausmann et al., 2008, Kato et al., 2008, Malathi et al., 2007, Malathi et al., 2010, Runge et al., 2014, Saito et al., 2008, Uzri and Gehrke, 2009). Although early studies suggested that in vitro transcribed 5′ppp single-stranded RNA (ssRNA) could induce IFN through RIG-I (Hornung et al., 2006, Kim et al., 2004), these results are now thought to be due to the generation of unexpected basepaired RNA products in *in vitro* transcription reactions, in addition to the expected ssRNA transcript (Cazenave and Uhlenbeck, 1994, Schmidt et al., 2009). More recent studies showed that there is a requirement for a region of blunt dsRNA directly adjacent to the 5′ppp (Schlee et al., 2009, Schmidt et al., 2009); these have since been backed up by structural studies (Kolakofsky et al., 2012, Kowalinski et al., 2011, Lu et al., 2010). The minimum length of the double-stranded region required to give 5′ppp-dependent IFN induction has been shown to range from 10 basepairs (bp) (Kohlway et al., 2013, Schmidt et al., 2009) to at least 19 bp (Schlee et al., 2009). Within this short dsRNA stretch, there has been shown to be some tolerance of wobble base-pairs, bulges and mismatches, as found in the ‘panhandle’ genome structures of single-stranded negative-sense RNA viruses (Marq et al., 2011, Schlee et al., 2009). It is noteworthy that there are a number of RNA structures, including 5′OH dsRNAs and 5′ppp dsRNAs with overhanging nucleotides, that can bind to RIG-I yet do not stimulate IFN induction (Hausmann et al., 2008, Lu et al., 2010, Marq et al., 2011, Schmidt et al., 2009, Takahasi et al., 2008). Indeed, dsRNA with a single overhanging 5′ppp nucleotide, such as those found in some arenavirus genomes, can potently inhibit IFN-β induction by a blunt-ended 5′ppp dsRNA, presumably by competing with blunt-ended 5′ppp dsRNA for RIG-I binding (Marq et al., 2011).

## 6 The influenza virus ‘panhandle’ as a RIG-I inducer

**Fig. 2:**
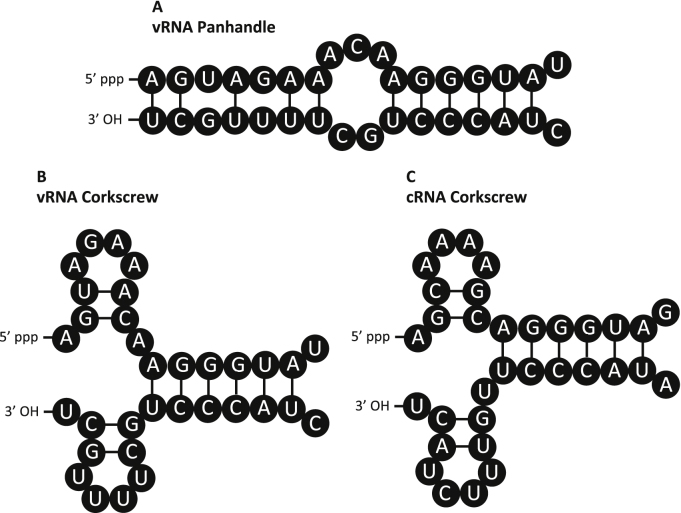
The panhandle and corkscrew structures of the influenza virus promoter. The panhandle (A) and corkscrew (B) conformations of influenza virus vRNA and the corkscrew conformation of cRNA (C) are shown. Sequences shown from segment 5 (NP) of the WSN strain of influenza virus.

The 5′ and 3′ termini of the influenza virus genome segments contain sequences of 13 and 12 nucleotides (nt) respectively that are highly conserved across segments and virus subtypes (with the exception of a U4C variation in the 3′ terminus of some genome segments) (Desselberger et al., 1980, Robertson, 1979). These sequences possess a partial inverted complementarity, and the influenza virus vRNA segments consequently have the potential to form a ‘panhandle’ structure (Fig. 2A) that is believed to act as the vRNA promoter (Fodor et al., 1994, Hsu et al., 1987, Tiley et al., 1994). NMR, FRET and enzymatic studies of short naked RNAs corresponding to the 3′ and 5′ termini have demonstrated the formation of a stable partial duplex of approximately 15 bp in length between the conserved termini and two to three additional segment-specific bases, through Watson–Crick and non-Watson–Crick basepairing (Fig. 2A) (Bae et al., 2001, Baudin et al., 1994, Cheong et al., 1996, Cheong et al., 1999, Hsu et al., 1987, Noble et al., 2011, Tomescu et al., 2014). Thus, the influenza virus panhandle has been suggested to be able to act as a potent RIG-I ligand by virtue of the 5′ppp being directly adjacent to a small stretch of partially double-stranded RNA. Although neither the formation of a panhandle structure within a full-length genome segment nor the contribution of terminal base-pairing to IFN induction by influenza virus have yet been directly demonstrated, *in vitro* transcription products corresponding to influenza virus segments are able to induce IFN when transfected into cells (Baum et al., 2010, Osterlund et al., 2012) suggesting that the influenza virus vRNAs possess an inherent ability to induce IFN. The observations that RNA extracted from influenza virus-infected cells, from purified influenza virions or from RNP reconstitutions activate the IFN response when transfected into cells in a RIG-I-dependent manner (Childs et al., 2012, Kato et al., 2008, Osterlund et al., 2012, Pichlmair et al., 2006, Rehwinkel et al., 2010) have also been used as evidence for IFN induction by the influenza panhandle. IFN induction by RNA extracted from infected cells is unaffected by RNase III treatment, which digests long dsRNAs into short fragments of 12–15 bp (Kato et al., 2008). This may be considered further support for IFN induction by the panhandle, since the base-paired stem would presumably be too short in length to be targeted by this enzyme.

While short free RNAs with the conserved 3′ and 5′ terminal sequences of vRNA forms a panhandle structure (Bae et al., 2001, Baudin et al., 1994, Cheong et al., 1996, Cheong et al., 1999, Hsu et al., 1987, Noble et al., 2011, Tomescu et al., 2014), evidence suggests that encapsidated polymerase-bound vRNA does not. Polymerase binding to the vRNA termini causes the formation of a secondary structure, the ‘corkscrew’ conformation, which has been shown by several studies to be critical for viral polymerase activity (Brownlee and Sharps, 2002, Flick and Hobom, 1999, Flick et al., 1996, Leahy et al., 2001a, Leahy et al., 2001b). In this structure, the very ends of the 5′ and 3′ termini do not associate but instead form intra-termini hairpin loops (Fig. 2B) (Flick et al., 1996). A highly sensitive single-molecule FRET assay was recently used to examine changes in the structure of short RNAs corresponding to the 5′ and 3′ termini of the genome segments. These RNAs form a partial dsRNA structure in the absence of viral polymerase but undergo a polymerase-dependent conformational change that is consistent with the formation of the corkscrew (Tomescu et al., 2014). The termini of free vRNA therefore form a dsRNA panhandle that is recognised and bound with high affinity by the viral polymerase, causing the vRNA to adopt the corkscrew conformation. Although the corkscrew permits some inter-strand basepairing, this structure would be unlikely to activate RIG-I since the terminal nucleotides do not bind each other to form dsRNA (Flick and Hobom, 1999, Fodor et al., 1995). Furthermore, recent structural determination of an influenza A virus polymerase in complex with the vRNA promoter appears to be incompatible with RIG-I binding of polymerase-associated RNA, since the 5′ppp end of the vRNA is buried within the polymerase in a pocket formed by PA and PB1 (Pflug et al., 2014). In order for an influenza virus genome segment to activate RIG-I therefore, it is likely to exist in the panhandle conformation, which is suggestive of a free, unencapsidated RNA.

The number of vRNA molecules within an infected cell vastly outnumbers cRNA, with the proportion of vRNAs to cRNAs estimated at between 10:1 and 40:1, depending on the segment (Hatada et al., 1989, Mukaigawa et al., 1991). For this reason, much of the work on influenza virus RNA and IFN induction has focused on the vRNA. Given that the cRNA is an exact complement of the vRNA, the cRNA termini are also partially complementary and would thus be expected to form panhandle and corkscrew structures. Indeed, cRNPs form closed helical loop structures similar to vRNPs (York et al., 2013) and the formation of a corkscrew structure by the cRNA termini has been demonstrated (Fig. 2C) (Azzeh et al., 2001, Crow et al., 2004). It is possible that structural differences exist between the vRNA and cRNA promoter structures however, since the wobble basepairs at positions 3 and 5 in the vRNA promoter become mismatched in the cRNA promoter; indeed, the structure of the cRNA promoter has been suggested to be more unstable than that of the vRNA (Park et al., 2003) so cRNA and vRNA may differ in the efficiency with which they activate RIG-I.

## 7 Identification of natural influenza virus PAMPs

### 7.1 Identification of RIG-I ligands from infected cells

Naked viral RNAs generated by *in vitro* transcription reactions or by RNA extractions from infected cells have been shown to induce IFN, yet such free RNAs would not be expected to be generated during virus infections due to vRNA and cRNA encapsidation. Two studies therefore addressed the nature of the ‘genuine’ viral PAMPs responsible for activating RIG-I during influenza virus infections. Baum et al. (2010) immunoprecipitated RIG-I from cells infected with an NS1-deficient virus, extracted RIG-I-associated RNA and performed deep sequencing analyses to identify RIG-I ligands. Although sequences mapping to all genome segments could be detected in RIG-I immunoprecipitates, there was particular enrichment for reads mapping to the smallest viral segments M and NS. Furthermore, reads mapping to the PA and PB1 polymerase segments were particularly over-represented in the 5′ and 3′ regions, which is highly suggestive of RNAs derived from genome segments containing large internal deletions. This analysis did not determine whether the RIG-I associated RNAs were vRNA, cRNA or mRNA in nature, since the amplification method used did not permit the retention of strand orientation information.

Rehwinkel et al. (2010) used a different approach to address the same question. The authors demonstrated the formation of a trimolecular complex of RIG-I, an in vitro RNA transcript and NS1, and by purifying this complex, RNA with IFN-inducing activity could be isolated. A similar complex was postulated to form with the bona fide RIG-I agonist during influenza virus infection, permitting genuine PAMPs from infected cells to be co-precipitated with the NS1 protein. Consistent with this, RNA purified from NS1-immunoprecipitates induced expression from an IFN-β reporter gene in a RIG-I- and 5′ppp-dependent manner, and NS1-associated RNA was enriched for full-length vRNA from all eight segments (in addition to cRNA and mRNA from some segments). Furthermore, RIG-I immunoprecipitates also contained full-length vRNA and cRNA, supporting the authors’ conclusions that full-length genomes constitute the major PAMP in infected cells.

The identification of influenza virus RNA, and in particular vRNA, as ligands of RIG-I in these studies is not surprising, given that the vRNA panhandle possesses the characteristics required for RIG-I activation. What remains to be determined however is how the vRNA panhandle structure is able to form in infected cells, since replication of the virus genome is so closely coupled to encapsidation of the nascent RNA. Two scenarios can be envisaged regarding RIG-I activation during influenza virus infections. The first is that free vRNA, forming a panhandle, is generated erroneously at some point in a virus infection; if this is the case, when and how is this free RNA species generated? The second is that RIG-I can somehow recognise vRNA within the context of a vRNP. Since the polymerase obscures the genome termini, the latter of these scenarios would presumably require displacement of the polymerase from the vRNA promoter for RIG-I recognition. Interestingly, the individual polymerase subunits have been found to associate with RIG-I in an RNA-independent manner (Li et al., 2014); although these associations were not found to have clear implications for activation of the IFN response, it is possible that RIG-I recruitment to the vRNP through polymerase interactions could induce a conformational change in the vRNP structure that leads to formation and exposure of the panhandle.

### 7.2 The requirements for transcription and replication

**Fig. 3:**
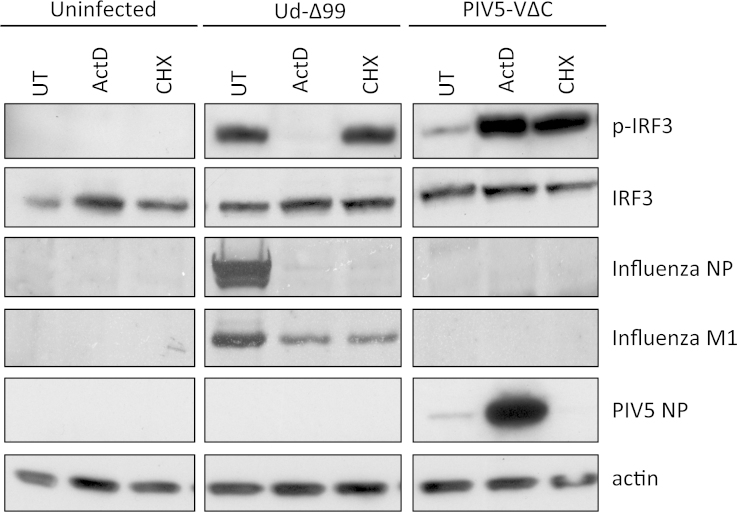
Activation of the IFN induction cascade by influenza virus is sensitive to actinomycin D but not cycloheximide. A549 cells were infected with 5 PFU/cell of an NS1-defective Udorn virus (Ud-Δ99; Jackson et al., 2010) or a DI-rich preparation of PIV5 (PIV5-VΔC vM2; Killip et al., 2011), in the presence of 1 μg/ml actinomycin D (ActD) or 50 μg/ml cycloheximide (CHX) or were left untreated (UT). At 16 h post-infection, cell lysates were prepared and probed for phospho-IRF3 (p-IRF3), total IRF3, viral proteins and actin.

Since influenza viruses replicate in the nucleus and RIG-I resides in the cytoplasm, incoming and progeny genomes traversing the cytoplasm are the most obvious candidates for triggering IFN induction, and there is some debate about the contribution of each of these to RIG-I activation by influenza viruses. Full-length genome replication requires ongoing viral protein synthesis due to the requirement for concurrent encapsidation by NP and newly synthesised polymerase (Honda et al., 1988, Jorba et al., 2009, Shapiro and Krug, 1988, Vreede et al., 2004, York et al., 2013); the translation inhibitor cycloheximide therefore prevents cRNP and progeny vRNP accumulation (and subsequently secondary viral transcription) in infected cells (Barrett et al., 1979, Hatada et al., 1989, Vreede et al., 2004). Rehwinkel et al. (2010) reported that no RNA capable of stimulating the IFN-β promoter could be extracted from infected cells in the presence of cycloheximide. Furthermore, they used an RNP reconstitution method to demonstrate IFN induction by reconstitutions with a transcription-defective but not a replication-defective viral polymerase and thus concluded that viral genome replication was required to stimulate IFN expression (Rehwinkel et al., 2010). However, recent studies have demonstrated IRF3 activation and transcription of both IFN-β and ISG56 genes in conditions where progeny vRNA synthesis is inhibited, either by cycloheximide treatment or using siRNA to NP (Fig. 3) (Killip et al., 2014, Osterlund et al., 2012, Weber et al., 2013).

Incoming viral nucleocapsids have been implicated in RIG-I activation by members of the Bunyaviridae, which also possess segmented negative-sense RNA genomes with a 5′ppp panhandle structure (Weber et al., 2013); activation of the IFN induction cascade in the presence of cycloheximide would be consistent with this also being the case for influenza virus infections. Indeed, a recent study has reported the association of vRNPs with RIG-I and MAVS at the mitochondrion at 3 h post-infection (Liedmann et al., 2014). However, several studies have indicated that incoming influenza A vRNPs are not sufficient to induce IFN during infection of epithelial cells and monocyte-derived DCs (moDCs), and that viral RNA synthesis is required (Crotta et al., 2013, Killip et al., 2014, Osterlund et al., 2012). Efficient inhibition of IRF3 and NF-kB activation by the cellular transcription inhibitors actinomycin D and alpha-amanitin in influenza-infected cells strongly suggests that incoming genomes do not function as a major PAMP in lung epithelial cells (Fig. 3) (Killip et al., 2014). These drugs do not affect the transport of incoming vRNPs to the nucleus, but potently inhibit viral transcription (because of the requirement for cellular transcripts for priming viral mRNA synthesis) and cRNP and vRNP generation (due to inhibition of de novo NP and polymerase synthesis). Virus-mediated IRF3 activation is also inhibited by 5,6-dichloro-1-β-d-ribofuranosyl-benzimidazole (DRB) treatment (Killip et al., 2014), which permits viral transcription but impedes the export of viral transcripts from the nucleus (Amorim et al., 2007, Chan et al., 2006). The effects of these drugs on IRF3 activation were specific to influenza viruses, since they have no effect on activation of the IFN induction cascade by the dsRNA analogue poly(I:C) or by paramyxoviruses (Fig. 3) (Killip et al., 2014) which replicate in the cytoplasm and do not rely on cellular transcription (Lamb and Parks, 2013). Moreover, expression of type I and type III IFNs following influenza A virus infection correlates with the accumulation of viral RNAs (Osterlund et al., 2012) and can be completely abrogated following inactivation of the virus by heat or UV treatment (Crotta et al., 2013, Osterlund et al., 2012). The above studies have been limited to a relatively small number of influenza A virus strains, so it is conceivable that different influenza A virus strains may vary in their capacity to be recognised by RIG-I during vRNP entry. Interestingly, influenza B viruses elicit a much more rapid activation of IRF3 than influenza A virus and this activation is insensitive to UV treatment (Osterlund et al., 2012) indicating that the incoming genomes of these different virus genera may differ in their ability to be recognised by PRRs.

Taken together, these observations strongly suggest that the generation of the major influenza A virus PAMPs requires the synthesis and nuclear export of an RNA product or products from incoming genomes, and that these RNAs can be generated even in conditions where cRNP and vRNP accumulation is impaired. Nevertheless, it is likely that distinct PAMPs are generated at different stages of the virus life cycle; thus, a minority of incoming influenza A virus genomes may contribute to IFN induction at very early points post-infection, with viral polymerase products (including, but not limited to, progeny genomes) functioning as a more significant PAMP population later in infection. The nature of the PAMPs that are generated in the presence of cycloheximide but not in the presence of cellular transcription inhibitors has not yet been elucidated, and although the sensitivity of IRF3 activation to viral transcription inhibitors may superficially suggest that viral mRNAs can function as IFN inducers, the fact that influenza virus mRNAs are 5′ capped and polyadenylated like host mRNAs makes this unlikely. Moreover, viral mRNAs cannot be detected in RIG-I immunoprecipitations from infected cells, the immunostimulatory activity of RIG-I associated RNA is sensitive to calf intestinal phosphatase treatment (which removes 5′ phosphates), and RNA capable of inducing IFN is still generated in RNP reconstitutions containing a transcription-defective viral polymerase (Rehwinkel et al., 2010). It is of note that actinomycin D and alpha-amanitin can also affect the nucleocytoplasmic export of viral mRNAs (Amorim et al., 2007, Killip et al., 2014, Mahy et al., 1980) so may also affect export of other viral RNA species; in actinomycin D and alpha-amanitin treatment conditions therefore, viral PAMPs may be retained in the nucleus and thus hidden from RIG-I. It has been suggested that cRNA synthesis from input vRNP templates does not require viral protein synthesis for the initiation of replication per se, but that *de novo* synthesised polymerase and NP are required to stabilise the cRNA and prevent its degradation by host nucleases (Vreede et al., 2004, Vreede et al., 2011). Unencapsidated, unstable cRNAs would therefore be expected to be generated in conditions where polymerase and NP synthesis has been blocked by transcription or translation inhibitors, and these RNAs may be able to activate RIG-I if they were able to reach the cytoplasm.

### 7.3 Defective genomes and IFN induction by influenza virus

The observations that influenza viruses only activate the IFN response in a fraction of infected cells with both wild-type and NS1-defective viruses (Fig. 1) (Chen et al., 2010, Kallfass et al., 2013) strongly suggest that activation of the IFN induction cascade is not associated with ‘normal’ virus replication, which is perhaps not surprising considering the nuclear localisation of replication and the efficient encapsidation of full-length genomes. The link between the presence of ‘abnormal’ DI genomes and IFN induction by non-segmented negative-strand RNA viruses, including the Paramyxoviridae and Rhabdoviridae, is well known. DI genomes possess deletions that render a DI virus particle unable to complete a full replication cycle due to the absence of the coding region for one or more viral factor that is essential for replication; DI viruses are therefore only able to replicate when a co-infecting non-defective virus is present to supplement the missing viral factor/s. Furthermore, DIs interfere with the multiplication of non-defective virus by competing with non-DI genomes for replication and packaging (reviewed in Marriott and Dimmock, 2010). Thus, although DI genomes are considered non-infectious in terms of their inability to generate viral progeny, they can still exert biological effects in host cells. In particular, IFN induction by paramyxoviruses and rhabdoviruses has been associated with ‘copyback’ or ‘snapback’ genome structures that are generated during template-switching of the viral RNA-dependent RNA polymerase between strands of opposite polarity (Baum et al., 2010, Johnston, 1981, Killip et al., 2011, Killip et al., 2013, Marcus and Gaccione, 1989, Shingai et al., 2007, Strahle et al., 2006). These structures possess often considerable stretches of dsRNA adjacent to a 5′ppp and are thus very effective RIG-I ligands (Baum et al., 2010, Strahle et al., 2007). Indeed, studies of IFN induction in single cells infected with a paramyxovirus lacking an IFN antagonist have indicated that the IFN-β promoter is not activated by the normal paramyxovirus life cycle, and that only DI-rich virus preparations activated the IFN-β promoter in a majority of cells (Killip et al., 2011). It is as yet unclear whether DI genomes themselves are responsible for inducing IFN, since they are encapsidated into stable nucleocapsids (Strahle et al., 2006) and should therefore be hidden from recognition by RIG-I. DI-rich paramyxovirus preparations are potent activators of IRF3 even when progeny genome synthesis has been inhibited by cycloheximide (Killip et al., 2012); however, unlike the situation for influenza viruses, the requirements for RNA synthesis to IFN induction by paramyxovirus DI genomes cannot easily be studied using inhibitors of cellular transcription, so it remains to be determined whether input DI genomes are sufficient for this process.

**Fig. 4:**
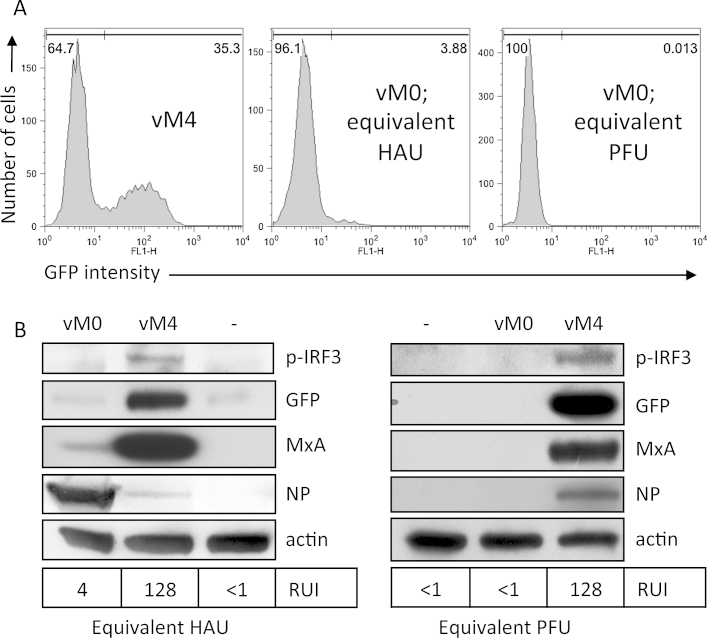
DIs in influenza virus preparations correlate with an enhanced ability to activate the interferon system. DI-preparations of PR8 were obtained by four sequential high multiplicity passages of the vM0 virus through MDCK cells. The total number of virus particles (haemagglutination units; HAU) and the plaque-forming titre (plaque-forming units; PFU) were obtained by haemagglutination assay and plaque assay respectively. A549 cells expressing GFP under the control of an IFN-β promoter (A549/pr(IFN-β).GFP; Chen et al., 2010) were infected with the low DI (vM0) or DI-rich (vM4) preparations of PR8 at equivalent infectious titre (0.3 PFU/cell) or an equivalent number of total virus particles (1 × 10^4^ HAU/cell). At 16 h post-infection, cells were trypsinised, fixed and analysed by flow cytometry for GFP expression (A). Duplicate monolayers were harvested for immunoblotting (B); lysates were probed for markers of activation of the interferon system (phospho-IRF3, GFP and MxA), viral NP expression and actin (as a loading control). The IFN present in culture media was estimated by CPE-reduction bio-assay, and the relative units of IFN (RUI) for each condition are indicated beneath the immunoblot panels.

Influenza virus readily generates DI genomes; DI influenza viruses were first identified in the 1950s as ‘incomplete’ non-infectious viruses that were selected for upon serial passage of the virus in embryonated chicken eggs at high multiplicity (Von Magnus, 1951a, Von Magnus, 1951b) and they have been described in many studies since (reviewed in Dimmock and Easton, 2014, Nayak, 1980). In contrast to non-segmented negative-strand RNA viruses, copyback structures are not thought to be generated during influenza virus replication. Instead, influenza DI viruses appear to be limited to internal deletion RNAs that retain the 5′ and 3′ termini of genome segment from which they are derived. Influenza virus DIs can arise from all genome segments, but appear to be more readily detectable from PB2, PB1 and PA segments. The generation of DIs has long been thought of as an artificial laboratory/tissue-culture artefact in laboratory-prepared and egg-derived virus stocks, and their relevance to genuine virus infections *in vivo* has been questioned. However, several recent studies have suggested an important role for DIs in natural influenza virus infections and in disease outcome. Internal deletion DI viruses similar to those identified *in vitro* have been detected in samples from infected mice (Tapia et al., 2013), poultry (Jonges et al., 2014) and humans (Jonges et al., 2014, Saira et al., 2013). Furthermore, DIs are generated *de novo* during *in vivo* infections (Tapia et al., 2013) and the transmission of DI viruses between human patients has been described (Saira et al., 2013). Evidence is accumulating for an important role of influenza virus DIs in IFN induction. Following a quantitative analysis of different subpopulations of an influenza virus, Marcus et al. (2005) concluded that IFN-inducing particles are not productively infectious. DI RNA was directly implicated in IFN induction by a study that identified subgenomic RNAs derived from PA and PB1 segments in RIG-I immunoprecipitates from influenza virus-infected cells (Baum et al., 2010). Subsequently, studies have correlated the IFN-inducing phenotype of influenza viruses with a propensity to generate or accumulate high numbers of DI genomes in tissue culture (Frensing et al., 2014, Ngunjiri et al., 2013, Perez-Cidoncha et al., 2014). Perez-Cidoncha and colleagues passaged influenza virus through IFN-resistant cells in order to select for mutants that were impaired in their ability to prevent IFN induction or signalling. By this method, a number of mutant viruses were obtained that were stronger IFN inducers than wild-type virus and several of these IFN-inducing mutants were found to accumulate large numbers of DI genomes in infected cells (Perez-Cidoncha et al., 2014). We have studied the link between influenza virus DIs and IFN induction in tissue culture and found that a preparation of PR8 virus that has been passaged so as to enrich for DI content is a much more efficient activator of the IFN response than our starting virus stock, when cells are infected with either an equivalent amount of total virus particles or plaque-forming virus particles (Fig. 4). Recent *in vivo* comparisons of the effects of DI-poor or DI-rich influenza virus preparations have shown that the presence of high numbers of DIs is associated with enhanced IFN-β expression in the lungs of infected mice and a reduction in animal morbidity (Tapia et al., 2013). Moreover, a preparation of influenza virus composed of a single DI species generated by reverse genetics (a internal deletion DI derived from segment 1, Dimmock et al., 2008) protects mice against infections by heterologous influenza B virus and paramyxovirus infections (Easton et al., 2011, Scott et al., 2011). This protection was impaired in mice lacking a functional type I IFN receptor, indicating that this protective effect is mediated at least in part by activation of the IFN response (Easton et al., 2011, Scott et al., 2011). What remains unclear is why influenza virus DI RNAs should be more effective at inducing IFN than non-defective, full-length genome segments. Due to the requirements for certain sequences at each end of the genome segment for packaging into virions (Gerber et al., 2014), internal deletion RNAs possess identical 5′ and 3′ termini to non-defective, full-length genomic RNAs and would not therefore be expected to differ in their inherent ability to bind RIG-I. The ability of DI viruses to induce IFN may simply be due to a faster replication rate of DI RNAs than full-length genomes because of their smaller size. Alternatively, DI-mediated interference with viral polymerase and NP expression may lead to a reduction in polymerase-imposed shut-off of cellular gene expression (Ngunjiri et al., 2012) or affect the efficiency of cRNA and vRNA encapsidation. Furthermore, DI-mediated interference may reduce NS1 expression, thereby contributing to IFN induction by limiting IFN antagonism by the virus. This occurs in DI-rich preparations of the paramyxovirus parainfluenza virus 5, where DIs simultaneously stimulate the IFN response and interfere with the expression of the V protein (the viral IFN antagonist) from co-infecting non-defective virus; as a result, the V protein only accumulates in infected cells after the IFN induction cascade has already been activated (Killip et al., 2013). Perhaps smaller segments of genomic RNA, such as the internal deletion RNAs and NS and M segment RNAs identified in RIG-I immunoprecipitates (Baum et al., 2010), form a panhandle more readily than full-length RNA when they are unencapsidated, or DI RNPs may be less stable than full-length RNPs and thus more prone to releasing free RNA. It is interesting to note that very small flu genome templates can be replicated in the absence of NP (Resa-Infante et al., 2010, Turrell et al., 2013); thus, it is a possibility that small DI RNAs could be replicated without being concurrently encapsidated and that these unencapsidated replication products could activate RIG-I.

## 8 Concluding remarks

Innate immune responses, including the IFNs, play a critical role in determining the pathogenicity and outcome of an influenza virus infection: efficient activation of the IFN system early in infection effectively restricts viral replication and eliminates the virus, while excessive activation of innate immune responses actually increases tissue damage in the host. Here, we have outlined recent advances in the study of the IFN induction by influenza viruses and the identity of the PAMPs produced during influenza virus infection; however, several unanswered questions remain. Assuming the influenza virus panhandle is recognised by RIG-I during infection, it is unclear how this RNA structure is able to form in infected cells: if the panhandle is formed by free unencapsidated RNA, how and when in the virus life cycle is this RNA generated? Conversely, if the panhandle structure can be formed by RNA encapsidated into RNPs, how are the polymerase and/or NP displaced in order to expose the genome termini? What are the viral PAMPs that are generated in conditions when progeny genome synthesis has been blocked? What are the reasons for the cell-to-cell differences in activation of the IFN promoter during influenza virus infections *in vitro* and *in vivo*? Lastly, why are influenza DIs better able to induce IFN than non-defective virus? Multiple factors are likely to influence IFN induction by influenza virus during an infection, including the rate of virus replication, its ability to actively antagonise IFN induction, the rate of DI generation by the viral polymerase and also factors conferred by the host; by gaining a broader comprehension of all of these contributing elements, we can more fully understand the interactions between influenza virus and the IFN system and apply this understanding to the development of novel anti-influenza therapies.

## References

[bib0005] Amorim M.J., Read E.K., Dalton R.M., Medcalf L., Digard P. (2007). Nuclear export of influenza A virus mRNAs requires ongoing RNA polymerase II activity. Traffic.

[bib0010] Azzeh M., Flick R., Hobom G. (2001). Functional analysis of the influenza A virus cRNA promoter and construction of an ambisense transcription system. Virology.

[bib0015] Bae S.H., Cheong H.K., Lee J.H., Cheong C., Kainosho M., Choi B.S. (2001). Structural features of an influenza virus promoter and their implications for viral RNA synthesis. Proc. Natl. Acad. Sci. U. S. A..

[bib0020] Barrett T., Wolstenholme A.J., Mahy B.W. (1979). Transcription and replication of influenza virus RNA. Virology.

[bib0025] Baskin C.R., Bielefeldt-Ohmann H., Tumpey T.M., Sabourin P.J., Long J.P., Garcia-Sastre A., Tolnay A.E., Albrecht R., Pyles J.A., Olson P.H., Aicher L.D., Rosenzweig E.R., Murali-Krishna K., Clark E.A., Kotur M.S., Fornek J.L., Proll S., Palermo R.E., Sabourin C.L., Katze M.G. (2009). Early and sustained innate immune response defines pathology and death in nonhuman primates infected by highly pathogenic influenza virus. Proc. Natl. Acad. Sci. U. S. A..

[bib0030] Baudin F., Bach C., Cusack S., Ruigrok R.W. (1994). Structure of influenza virus RNP. I. Influenza virus nucleoprotein melts secondary structure in panhandle RNA and exposes the bases to the solvent. EMBO J..

[bib0035] Baum A., Sachidanandam R., Garcia-Sastre A. (2010). Preference of RIG-I for short viral RNA molecules in infected cells revealed by next-generation sequencing. Proc. Natl. Acad. Sci. U. S. A..

[bib0040] Binder M., Eberle F., Seitz S., Mucke N., Huber C.M., Kiani N., Kaderali L., Lohmann V., Dalpke A., Bartenschlager R. (2011). Molecular mechanism of signal perception and integration by the innate immune sensor retinoic acid-inducible gene-I (RIG-I). J. Biol. Chem..

[bib0045] Bonin M., Oberstrass J., Lukacs N., Ewert K., Oesterschulze E., Kassing R., Nellen W. (2000). Determination of preferential binding sites for anti-dsRNA antibodies on double-stranded RNA by scanning force microscopy. RNA.

[bib0050] Brass A.L., Huang I.C., Benita Y., John S.P., Krishnan M.N., Feeley E.M., Ryan B.J., Weyer J.L., van der Weyden L., Fikrig E., Adams D.J., Xavier R.J., Farzan M., Elledge S.J. (2009). The IFITM proteins mediate cellular resistance to influenza A H1N1 virus, West Nile virus, and dengue virus. Cell.

[bib0055] Brownlee G.G., Sharps J.L. (2002). The RNA polymerase of influenza a virus is stabilized by interaction with its viral RNA promoter. J. Virol..

[bib0060] Cazenave C., Uhlenbeck O.C. (1994). RNA template-directed RNA synthesis by T7 RNA polymerase. Proc. Natl. Acad. Sci. U. S. A..

[bib0065] Chan A.Y., Vreede F.T., Smith M., Engelhardt O.G., Fodor E. (2006). Influenza virus inhibits RNA polymerase II elongation. Virology.

[bib0070] Chen S., Short J.A., Young D.F., Killip M.J., Schneider M., Goodbourn S., Randall R.E. (2010). Heterocellular induction of interferon by negative-sense RNA viruses. Virology.

[bib0075] Cheong H.K., Cheong C., Choi B.S. (1996). Secondary structure of the panhandle RNA of influenza virus A studied by NMR spectroscopy. Nucleic Acids Res..

[bib0080] Cheong H.K., Cheong C., Lee Y.S., Seong B.L., Choi B.S. (1999). Structure of influenza virus panhandle RNA studied by NMR spectroscopy and molecular modeling. Nucleic Acids Res..

[bib0085] Cheung C.Y., Poon L.L., Lau A.S., Luk W., Lau Y.L., Shortridge K.F., Gordon S., Guan Y., Peiris J.S. (2002). Induction of proinflammatory cytokines in human macrophages by influenza A (H5N1) viruses: a mechanism for the unusual severity of human disease. Lancet.

[bib0090] Childs K., Randall R., Goodbourn S. (2012). Paramyxovirus V proteins interact with the RNA Helicase LGP2 to inhibit RIG-I-dependent interferon induction. J. Virol..

[bib0095] Childs K.S., Randall R.E., Goodbourn S. (2013). LGP2 plays a critical role in sensitizing mda-5 to activation by double-stranded RNA. PLoS ONE.

[bib0100] Crotta S., Davidson S., Mahlakoiv T., Desmet C.J., Buckwalter M.R., Albert M.L., Staeheli P., Wack A. (2013). Type I and type III interferons drive redundant amplification loops to induce a transcriptional signature in influenza-infected airway epithelia. PLoS Pathog..

[bib0105] Crow M., Deng T., Addley M., Brownlee G.G. (2004). Mutational analysis of the influenza virus cRNA promoter and identification of nucleotides critical for replication. J. Virol..

[bib0110] Davis W.G., Bowzard J.B., Sharma S.D., Wiens M.E., Ranjan P., Gangappa S., Stuchlik O., Pohl J., Donis R.O., Katz J.M., Cameron C.E., Fujita T., Sambhara S. (2012). The 3′ untranslated regions of influenza genomic sequences are 5′PPP-independent ligands for RIG-I. PLoS ONE.

[bib0115] Desai T.M., Marin M., Chin C.R., Savidis G., Brass A.L., Melikyan G.B. (2014). IFITM3 restricts influenza A virus entry by blocking the formation of fusion pores following virus-endosome hemifusion. PLoS Pathog..

[bib0120] Desselberger U., Racaniello V.R., Zazra J.J., Palese P. (1980). The 3′ and 5′-terminal sequences of influenza A, B and C virus RNA segments are highly conserved and show partial inverted complementarity. Gene.

[bib0125] Diebold S.S., Kaisho T., Hemmi H., Akira S., Reis e Sousa C. (2004). Innate antiviral responses by means of TLR7-mediated recognition of single-stranded RNA. Science.

[bib0130] Dimmock N.J., Easton A.J. (2014). Defective interfering influenza virus RNAs: time to reevaluate their clinical potential as broad-spectrum antivirals?. J. Virol..

[bib0135] Dimmock N.J., Rainsford E.W., Scott P.D., Marriott A.C. (2008). Influenza virus protecting RNA: an effective prophylactic and therapeutic antiviral. J. Virol..

[bib0140] Donelan N.R., Basler C.F., Garcia-Sastre A. (2003). A recombinant influenza A virus expressing an RNA-binding-defective NS1 protein induces high levels of beta interferon and is attenuated in mice. J. Virol..

[bib0145] Dudek S.E., Wixler L., Nordhoff C., Nordmann A., Anhlan D., Wixler V., Ludwig S. (2011). The influenza virus PB1-F2 protein has interferon antagonistic activity. Biol. Chem..

[bib0150] Easton A.J., Scott P.D., Edworthy N.L., Meng B., Marriott A.C., Dimmock N.J. (2011). A novel broad-spectrum treatment for respiratory virus infections: influenza-based defective interfering virus provides protection against pneumovirus infection in vivo. Vaccine.

[bib0155] Egorov A., Brandt S., Sereinig S., Romanova J., Ferko B., Katinger D., Grassauer A., Alexandrova G., Katinger H., Muster T. (1998). Transfectant influenza A viruses with long deletions in the NS1 protein grow efficiently in Vero cells. J. Virol..

[bib0160] Engel D.A. (2013). The influenza virus NS1 protein as a therapeutic target. Antivir. Res..

[bib0165] Everitt A.R., Clare S., Pertel T., John S.P., Wash R.S., Smith S.E., Chin C.R., Feeley E.M., Sims J.S., Adams D.J., Wise H.M., Kane L., Goulding D., Digard P., Anttila V., Baillie J.K., Walsh T.S., Hume D.A., Palotie A., Xue Y., Colonna V., Tyler-Smith C., Dunning J., Gordon S.B., Smyth R.L., Openshaw P.J., Dougan G., Brass A.L., Kellam P. (2012). IFITM3 restricts the morbidity and mortality associated with influenza. Nature.

[bib0170] Feeley E.M., Sims J.S., John S.P., Chin C.R., Pertel T., Chen L.M., Gaiha G.D., Ryan B.J., Donis R.O., Elledge S.J., Brass A.L. (2011). IFITM3 inhibits influenza A virus infection by preventing cytosolic entry. PLoS Pathog..

[bib0175] Flick R., Hobom G. (1999). Interaction of influenza virus polymerase with viral RNA in the ‘corkscrew’ conformation. J. Gen. Virol..

[bib0180] Flick R., Neumann G., Hoffmann E., Neumeier E., Hobom G. (1996). Promoter elements in the influenza vRNA terminal structure. RNA.

[bib0185] Fodor E. (2013). The RNA polymerase of influenza a virus: mechanisms of viral transcription and replication. Acta Virol..

[bib0190] Fodor E., Pritlove D.C., Brownlee G.G. (1994). The influenza virus panhandle is involved in the initiation of transcription. J. Virol..

[bib0195] Fodor E., Pritlove D.C., Brownlee G.G. (1995). Characterization of the RNA-fork model of virion RNA in the initiation of transcription in influenza A virus. J. Virol..

[bib0200] Fortes P., Beloso A., Ortin J. (1994). Influenza virus NS1 protein inhibits pre-mRNA splicing and blocks mRNA nucleocytoplasmic transport. EMBO J..

[bib0205] Frensing T., Pflugmacher A., Bachmann M., Peschel B., Reichl U. (2014). Impact of defective interfering particles on virus replication and antiviral host response in cell culture-based influenza vaccine production. Appl. Microbiol. Biotechnol..

[bib0210] Gack M.U. (2014). Mechanisms of RIG-I-like receptor activation and manipulation by viral pathogens. J. Virol..

[bib0215] Gack M.U., Albrecht R.A., Urano T., Inn K.S., Huang I.C., Carnero E., Farzan M., Inoue S., Jung J.U., Garcia-Sastre A. (2009). Influenza A virus NS1 targets the ubiquitin ligase TRIM25 to evade recognition by the host viral RNA sensor RIG-I. Cell Host Microbe.

[bib0220] Gack M.U., Shin Y.C., Joo C.H., Urano T., Liang C., Sun L., Takeuchi O., Akira S., Chen Z., Inoue S., Jung J.U. (2007). TRIM25 RING-finger E3 ubiquitin ligase is essential for RIG-I-mediated antiviral activity. Nature.

[bib0225] Gao S., von der Malsburg A., Dick A., Faelber K., Schroder G.F., Haller O., Kochs G., Daumke O. (2011). Structure of myxovirus resistance protein a reveals intra- and intermolecular domain interactions required for the antiviral function. Immunity.

[bib0230] Garcia-Sastre A. (2011). Induction and evasion of type I interferon responses by influenza viruses. Virus Res..

[bib0235] Garcia-Sastre A., Egorov A., Matassov D., Brandt S., Levy D.E., Durbin J.E., Palese P., Muster T. (1998). Influenza A virus lacking the NS1 gene replicates in interferon-deficient systems. Virology.

[bib0240] Gerber M., Isel C., Moules V., Marquet R. (2014). Selective packaging of the influenza A genome and consequences for genetic reassortment. Trends Microbiol..

[bib0245] Goubau D., Schlee M., Deddouche S., Pruijssers A.J., Zillinger T., Goldeck M., Schuberth C., Van der Veen A.G., Fujimura T., Rehwinkel J., Iskarpatyoti J.A., Barchet W., Ludwig J., Dermody T.S., Hartmann G., Reis E.S.C. (2014). Antiviral immunity via RIG-I-mediated recognition of RNA bearing 5′-diphosphates. Nature.

[bib0250] Graef K.M., Vreede F.T., Lau Y.F., McCall A.W., Carr S.M., Subbarao K., Fodor E. (2010). The PB2 subunit of the influenza virus RNA polymerase affects virulence by interacting with the mitochondrial antiviral signaling protein and inhibiting expression of beta interferon. J. Virol..

[bib0255] Guillot L., Le Goffic R., Bloch S., Escriou N., Akira S., Chignard M., Si-Tahar M. (2005). Involvement of toll-like receptor 3 in the immune response of lung epithelial cells to double-stranded RNA and influenza A virus. J. Biol. Chem..

[bib0260] Hale B.G., Randall R.E., Ortin J., Jackson D. (2008). The multifunctional NS1 protein of influenza A viruses. J. Gen. Virol..

[bib0265] Haller O., Arnheiter H., Lindenmann J., Gresser I. (1980). Host gene influences sensitivity to interferon action selectively for influenza virus. Nature.

[bib0270] Hatada E., Hasegawa M., Mukaigawa J., Shimizu K., Fukuda R. (1989). Control of influenza virus gene expression: quantitative analysis of each viral RNA species in infected cells. J. Biochem..

[bib0275] Hausmann S., Marq J.B., Tapparel C., Kolakofsky D., Garcin D. (2008). RIG-I and dsRNA-induced IFNbeta activation. PLoS ONE.

[bib0280] Hay A.J., Lomniczi B., Bellamy A.R., Skehel J.J. (1977). Transcription of the influenza virus genome. Virology.

[bib0285] Hayman A., Comely S., Lackenby A., Murphy S., McCauley J., Goodbourn S., Barclay W. (2006). Variation in the ability of human influenza A viruses to induce and inhibit the IFN-beta pathway. Virology.

[bib0290] Hogner K., Wolff T., Pleschka S., Plog S., Gruber A.D., Kalinke U., Walmrath H.D., Bodner J., Gattenlohner S., Lewe-Schlosser P., Matrosovich M., Seeger W., Lohmeyer J., Herold S. (2013). Macrophage-expressed IFN-beta contributes to apoptotic alveolar epithelial cell injury in severe influenza virus pneumonia. PLoS Pathog..

[bib0295] Honda A., Ueda K., Nagata K., Ishihama A. (1988). RNA polymerase of influenza virus: role of NP in RNA chain elongation. J. Biochem..

[bib0300] Hornung V., Ellegast J., Kim S., Brzozka K., Jung A., Kato H., Poeck H., Akira S., Conzelmann K.K., Schlee M., Endres S., Hartmann G. (2006). 5′-Triphosphate RNA is the ligand for RIG-I. Science.

[bib0305] Hsu M.T., Parvin J.D., Gupta S., Krystal M., Palese P. (1987). Genomic RNAs of influenza viruses are held in a circular conformation in virions and in infected cells by a terminal panhandle. Proc. Natl. Acad. Sci. U. S. A..

[bib0310] Husser L., Alves M.P., Ruggli N., Summerfield A. (2011). Identification of the role of RIG-I, MDA-5 and TLR3 in sensing RNA viruses in porcine epithelial cells using lentivirus-driven RNA interference. Virus Res..

[bib0315] Ioannidis I., Ye F., McNally B., Willette M., Flano E. (2013). Toll-like receptor expression and induction of type I and type III interferons in primary airway epithelial cells. J. Virol..

[bib0320] Iwai A., Shiozaki T., Kawai T., Akira S., Kawaoka Y., Takada A., Kida H., Miyazaki T. (2010). Influenza A virus polymerase inhibits type I interferon induction by binding to interferon beta promoter stimulator 1. J. Biol. Chem..

[bib1325] Jackson D., Killip M.J., Galloway C.S., Russell R.J., Randall R.E. (2010). Loss of function of the influenza A virus NS1 protein promotes apoptosis but this is not due to a failure to activate phosphatidylinositol 3-kinase (PI3K). Virology.

[bib0325] Jewell N.A., Cline T., Mertz S.E., Smirnov S.V., Flano E., Schindler C., Grieves J.L., Durbin R.K., Kotenko S.V., Durbin J.E. (2010). Lambda interferon is the predominant interferon induced by influenza A virus infection in vivo. J. Virol..

[bib0330] Jewell N.A., Vaghefi N., Mertz S.E., Akter P., Peebles R.S., Bakaletz L.O., Durbin R.K., Flano E., Durbin J.E. (2007). Differential type I interferon induction by respiratory syncytial virus and influenza a virus in vivo. J. Virol..

[bib0335] Johnston M.D. (1981). The characteristics required for a Sendai virus preparation to induce high levels of interferon in human lymphoblastoid cells. J. Gen. Virol..

[bib0340] Jonges M., Welkers M.R., Jeeninga R.E., Meijer A., Schneeberger P., Fouchier R.A., de Jong M.D., Koopmans M. (2014). Emergence of the virulence-associated PB2 E627 K substitution in a fatal human case of highly pathogenic avian influenza virus A (H7N7) infection as determined by Illumina ultra-deep sequencing. J. Virol..

[bib0345] Jorba N., Coloma R., Ortin J. (2009). Genetic trans-complementation establishes a new model for influenza virus RNA transcription and replication. PLoS Pathog..

[bib0350] Kallfass C., Lienenklaus S., Weiss S., Staeheli P. (2013). Visualizing the beta interferon response in mice during infection with influenza A viruses expressing or lacking nonstructural protein 1. J. Virol..

[bib0355] Kaminski M.M., Ohnemus A., Cornitescu M., Staeheli P. (2012). Plasmacytoid dendritic cells and Toll-like receptor 7-dependent signalling promote efficient protection of mice against highly virulent influenza A virus. J. Gen. Virol..

[bib0360] Karpala A.J., Stewart C., McKay J., Lowenthal J.W., Bean A.G. (2011). Characterization of chicken Mda5 activity: regulation of IFN-beta in the absence of RIG-I functionality. J. Immunol..

[bib0365] Kash J.C., Tumpey T.M., Proll S.C., Carter V., Perwitasari O., Thomas M.J., Basler C.F., Palese P., Taubenberger J.K., Garcia-Sastre A., Swayne D.E., Katze M.G. (2006). Genomic analysis of increased host immune and cell death responses induced by 1918 influenza virus. Nature.

[bib0370] Kato H., Sato S., Yoneyama M., Yamamoto M., Uematsu S., Matsui K., Tsujimura T., Takeda K., Fujita T., Takeuchi O., Akira S. (2005). Cell type-specific involvement of RIG-I in antiviral response. Immunity.

[bib0375] Kato H., Takeuchi O., Mikamo-Satoh E., Hirai R., Kawai T., Matsushita K., Hiiragi A., Dermody T.S., Fujita T., Akira S. (2008). Length-dependent recognition of double-stranded ribonucleic acids by retinoic acid-inducible gene-I and melanoma differentiation-associated gene 5. J. Exp. Med..

[bib0380] Kato H., Takeuchi O., Sato S., Yoneyama M., Yamamoto M., Matsui K., Uematsu S., Jung A., Kawai T., Ishii K.J., Yamaguchi O., Otsu K., Tsujimura T., Koh C.S., Reis e Sousa C., Matsuura Y., Fujita T., Akira S. (2006). Differential roles of MDA5 and RIG-I helicases in the recognition of RNA viruses. Nature.

[bib0385] Khaitov M.R., Laza-Stanca V., Edwards M.R., Walton R.P., Rohde G., Contoli M., Papi A., Stanciu L.A., Kotenko S.V., Johnston S.L. (2009). Respiratory virus induction of alpha-, beta- and lambda-interferons in bronchial epithelial cells and peripheral blood mononuclear cells. Allergy.

[bib0390] Killip M.J., Smith M., Jackson D., Randall R.E. (2014). Activation of the interferon induction cascade by influenza a viruses requires viral RNA synthesis and nuclear export. J. Virol..

[bib0395] Killip M.J., Young D.F., Gatherer D., Ross C.S., Short J.A., Davison A.J., Goodbourn S., Randall R.E. (2013). Deep sequencing analysis of defective genomes of parainfluenza virus 5 and their role in interferon induction. J. Virol..

[bib0400] Killip M.J., Young D.F., Precious B.L., Goodbourn S., Randall R.E. (2012). Activation of the beta interferon promoter by paramyxoviruses in the absence of virus protein synthesis. J. Gen. Virol..

[bib0405] Killip M.J., Young D.F., Ross C.S., Chen S., Goodbourn S., Randall R.E. (2011). Failure to activate the IFN-beta promoter by a paramyxovirus lacking an interferon antagonist. Virology.

[bib0410] Kim D.H., Longo M., Han Y., Lundberg P., Cantin E., Rossi J.J. (2004). Interferon induction by siRNAs and ssRNAs synthesized by phage polymerase. Nat. Biotechnol..

[bib0415] Kochs G., Martinez-Sobrido L., Lienenklaus S., Weiss S., Garcia-Sastre A., Staeheli P. (2009). Strong interferon-inducing capacity of a highly virulent variant of influenza A virus strain PR8 with deletions in the NS1 gene. J. Gen. Virol..

[bib0420] Kohlway A., Luo D., Rawling D.C., Ding S.C., Pyle A.M. (2013). Defining the functional determinants for RNA surveillance by RIG-I. EMBO Rep..

[bib0425] Kolakofsky D., Kowalinski E., Cusack S. (2012). A structure-based model of RIG-I activation. RNA.

[bib0430] Kowalinski E., Lunardi T., McCarthy A.A., Louber J., Brunel J., Grigorov B., Gerlier D., Cusack S. (2011). Structural basis for the activation of innate immune pattern-recognition receptor RIG-I by viral RNA. Cell.

[bib0435] Koyama S., Ishii K.J., Kumar H., Tanimoto T., Coban C., Uematsu S., Kawai T., Akira S. (2007). Differential role of TLR- and RLR-signaling in the immune responses to influenza A virus infection and vaccination. J. Immunol..

[bib0440] Kumar H., Kawai T., Kato H., Sato S., Takahashi K., Coban C., Yamamoto M., Uematsu S., Ishii K.J., Takeuchi O., Akira S. (2006). Essential role of IPS-1 in innate immune responses against RNA viruses. J. Exp. Med..

[bib0445] Kuniyoshi K., Takeuchi O., Pandey S., Satoh T., Iwasaki H., Akira S., Kawai T. (2014). Pivotal role of RNA-binding E3 ubiquitin ligase MEX3C in RIG-I-mediated antiviral innate immunity. Proc. Natl. Acad. Sci. U. S. A..

[bib0450] Lamb R.A., Parks G.D., Knipe D.M., Howley P.M. (2013). Paramyxoviridae. Fields Virology.

[bib0455] Le Goffic R., Pothlichet J., Vitour D., Fujita T., Meurs E., Chignard M., Si-Tahar M. (2007). Cutting edge: influenza A virus activates TLR3-dependent inflammatory and RIG-I-dependent antiviral responses in human lung epithelial cells. J. Immunol..

[bib0460] Leahy M.B., Dobbyn H.C., Brownlee G.G. (2001). Hairpin loop structure in the 3′ arm of the influenza A virus virion RNA promoter is required for endonuclease activity. J. Virol..

[bib0465] Leahy M.B., Pritlove D.C., Poon L.L., Brownlee G.G. (2001). Mutagenic analysis of the 5′ arm of the influenza A virus virion RNA promoter defines the sequence requirements for endonuclease activity. J. Virol..

[bib0470] Lester S.N., Li K. (2014). Toll-like receptors in antiviral innate immunity. J. Mol. Biol..

[bib0475] Li K., Markosyan R.M., Zheng Y.M., Golfetto O., Bungart B., Li M., Ding S., He Y., Liang C., Lee J.C., Gratton E., Cohen F.S., Liu S.L. (2013). IFITM proteins restrict viral membrane hemifusion. PLoS Pathog..

[bib0480] Li W., Chen H., Sutton T., Obadan A., Perez D.R. (2014). Interactions between the influenza A virus RNA polymerase components and retinoic acid-inducible gene I. J. Virol..

[bib0485] Liedmann S., Hrincius E.R., Guy C., Anhlan D., Dierkes R., Carter R., Wu G., Staeheli P., Green D.R., Wolff T., McCullers J.A., Ludwig S., Ehrhardt C. (2014). Viral suppressors of the RIG-I-mediated interferon response are pre-packaged in influenza virions. Nat. Commun..

[bib0490] Liniger M., Summerfield A., Zimmer G., McCullough K.C., Ruggli N. (2012). Chicken cells sense influenza A virus infection through MDA5 and CARDIF signaling involving LGP2. J. Virol..

[bib0495] Loo Y.M., Fornek J., Crochet N., Bajwa G., Perwitasari O., Martinez-Sobrido L., Akira S., Gill M.A., Garcia-Sastre A., Katze M.G., Gale M. (2008). Distinct RIG-I and MDA5 signaling by RNA viruses in innate immunity. J. Virol..

[bib0500] Lu C., Xu H., Ranjith-Kumar C.T., Brooks M.T., Hou T.Y., Hu F., Herr A.B., Strong R.K., Kao C.C., Li P. (2010). The structural basis of 5′ triphosphate double-stranded RNA recognition by RIG-I C-terminal domain. Structure.

[bib0505] Lund J.M., Alexopoulou L., Sato A., Karow M., Adams N.C., Gale N.W., Iwasaki A., Flavell R.A. (2004). Recognition of single-stranded RNA viruses by Toll-like receptor 7. Proc. Natl. Acad. Sci. U. S. A..

[bib0510] Luo D., Ding S.C., Vela A., Kohlway A., Lindenbach B.D., Pyle A.M. (2011). Structural insights into RNA recognition by RIG-I. Cell.

[bib0515] Mahy B.W., Barrett T., Briedis D.J., Brownson J.M., Wolstenholme A.J. (1980). Influence of the host cell on influenza virus replication. Philos. Trans. R. Soc. Lond. B: Biol. Sci..

[bib0520] Malathi K., Dong B., Gale M., Silverman R.H. (2007). Small self-RNA generated by RNase L amplifies antiviral innate immunity. Nature.

[bib0525] Malathi K., Saito T., Crochet N., Barton D.J., Gale M., Silverman R.H. (2010). RNase L releases a small RNA from HCV RNA that refolds into a potent PAMP. RNA.

[bib0530] Marcus P.I., Gaccione C. (1989). Interferon induction by viruses. XIX. Vesicular stomatitis virus – New Jersey: high multiplicity passages generate interferon-inducing, defective-interfering particles. Virology.

[bib0535] Marcus P.I., Rojek J.M., Sekellick M.J. (2005). Interferon induction and/or production and its suppression by influenza A viruses. J. Virol..

[bib0540] Mark G.E., Taylor J.M., Broni B., Krug R.M. (1979). Nuclear accumulation of influenza viral RNA transcripts and the effects of cycloheximide, actinomycin D, and alpha-amanitin. J. Virol..

[bib0545] Marq J.B., Hausmann S., Veillard N., Kolakofsky D., Garcin D. (2011). Short double-stranded RNAs with an overhanging 5′ ppp-nucleotide, as found in arenavirus genomes, act as RIG-I decoys. J. Biol. Chem..

[bib0550] Marriott A.C., Dimmock N.J. (2010). Defective interfering viruses and their potential as antiviral agents. Rev. Med. Virol..

[bib0555] Mayer A.K., Muehmer M., Mages J., Gueinzius K., Hess C., Heeg K., Bals R., Lang R., Dalpke A.H. (2007). Differential recognition of TLR-dependent microbial ligands in human bronchial epithelial cells. J. Immunol..

[bib0560] Mena I., Jambrina E., Albo C., Perales B., Ortin J., Arrese M., Vallejo D., Portela A. (1999). Mutational analysis of influenza A virus nucleoprotein: identification of mutations that affect RNA replication. J. Virol..

[bib0565] Momose F., Basler C.F., O’Neill R.E., Iwamatsu A., Palese P., Nagata K. (2001). Cellular splicing factor RAF-2p48/NPI-5/BAT1/UAP56 interacts with the influenza virus nucleoprotein and enhances viral RNA synthesis. J. Virol..

[bib0570] Mordstein M., Neugebauer E., Ditt V., Jessen B., Rieger T., Falcone V., Sorgeloos F., Ehl S., Mayer D., Kochs G., Schwemmle M., Gunther S., Drosten C., Michiels T., Staeheli P. (2010). Lambda interferon renders epithelial cells of the respiratory and gastrointestinal tracts resistant to viral infections. J. Virol..

[bib0575] Mukaigawa J., Hatada E., Fukuda R., Shimizu K. (1991). Involvement of the influenza A virus PB2 protein in the regulation of viral gene expression. J. Gen. Virol..

[bib0580] Naito T., Kiyasu Y., Sugiyama K., Kimura A., Nakano R., Matsukage A., Nagata K. (2007). An influenza virus replicon system in yeast identified Tat-SF1 as a stimulatory host factor for viral RNA synthesis. Proc. Natl. Acad. Sci. U. S. A..

[bib0585] Nayak D.P. (1980). Defective interfering influenza viruses. Annu. Rev. Microbiol..

[bib0590] Nemeroff M.E., Barabino S.M., Li Y., Keller W., Krug R.M. (1998). Influenza virus NS1 protein interacts with the cellular 30 kDa subunit of CPSF and inhibits 3′ end formation of cellular pre-mRNAs. Mol. Cell.

[bib0595] Ngunjiri J.M., Buchek G.M., Mohni K.N., Sekellick M.J., Marcus P.I. (2013). Influenza virus subpopulations: exchange of lethal H5N1 virus NS for H1N1 virus NS triggers de novo generation of defective-interfering particles and enhances interferon-inducing particle efficiency. J. Interferon Cytokine Res..

[bib0600] Ngunjiri J.M., Lee C.W., Ali A., Marcus P.I. (2012). Influenza virus interferon-inducing particle efficiency is reversed in avian and mammalian cells, and enhanced in cells co-infected with defective-interfering particles. J. Interferon Cytokine Res..

[bib0605] Noah D.L., Twu K.Y., Krug R.M. (2003). Cellular antiviral responses against influenza A virus are countered at the posttranscriptional level by the viral NS1A protein via its binding to a cellular protein required for the 3′ end processing of cellular pre-mRNAS. Virology.

[bib0610] Noble E., Mathews D.H., Chen J.L., Turner D.H., Takimoto T., Kim B. (2011). Biophysical analysis of influenza A virus RNA promoter at physiological temperatures. J. Biol. Chem..

[bib0615] Onoguchi K., Yoneyama M., Takemura A., Akira S., Taniguchi T., Namiki H., Fujita T. (2007). Viral infections activate types I and III interferon genes through a common mechanism. J. Biol. Chem..

[bib0620] Oshiumi H., Matsumoto M., Hatakeyama S., Seya T. (2009). Riplet/RNF135, a RING finger protein, ubiquitinates RIG-I to promote interferon-β induction during the early phase of viral infection. J. Biol. Chem..

[bib0625] Oshiumi H., Miyashita M., Inoue N., Okabe M., Matsumoto M., Seya T. (2010). The ubiquitin ligase Riplet is essential for RIG-I-dependent innate immune responses to RNA virus infection. Cell Host Microbe.

[bib0630] Osterlund P., Strengell M., Sarin L.P., Poranen M.M., Fagerlund R., Melen K., Julkunen I. (2012). Incoming influenza A virus evades early host recognition, while influenza B virus induces interferon expression directly upon entry. J. Virol..

[bib0635] Osterlund P.I., Pietila T.E., Veckman V., Kotenko S.V., Julkunen I. (2007). IFN regulatory factor family members differentially regulate the expression of type III IFN (IFN-lambda) genes. J. Immunol..

[bib0640] Park C.J., Bae S.H., Lee M.K., Varani G., Choi B.S. (2003). Solution structure of the influenza A virus cRNA promoter: implications for differential recognition of viral promoter structures by RNA-dependent RNA polymerase. Nucleic Acids Res..

[bib0645] Pavlovic J., Haller O., Staeheli P. (1992). Human and mouse Mx proteins inhibit different steps of the influenza virus multiplication cycle. J. Virol..

[bib0650] Perez-Cidoncha M., Killip M.J., Oliveros J.C., Asensio V.J., Fernandez Y., Bengoechea J.A., Randall R.E., Ortin J. (2014). An unbiased genetic screen reveals the polygenic nature of the influenza virus anti-interferon response. J. Virol..

[bib0655] Pflug A., Guilligay D., Reich S., Cusack S. (2014). Structure of influenza A polymerase bound to the viral RNA promoter. Nature.

[bib0660] Pichlmair A., Schulz O., Tan C.P., Naslund T.I., Liljestrom P., Weber F., Reis e Sousa C. (2006). RIG-I-mediated antiviral responses to single-stranded RNA bearing 5′-phosphates. Science.

[bib0665] Rand U., Rinas M., Schwerk J., Nohren G., Linnes M., Kroger A., Flossdorf M., Kaly-Kullai K., Hauser H., Hofer T., Koster M. (2012). Multi-layered stochasticity and paracrine signal propagation shape the type-I interferon response. Mol. Syst. Biol..

[bib0670] Randall R.E., Goodbourn S. (2008). Interferons and viruses: an interplay between induction, signalling, antiviral responses and virus countermeasures. J. Gen. Virol..

[bib0675] Rehwinkel J., Tan C.P., Goubau D., Schulz O., Pichlmair A., Bier K., Robb N., Vreede F., Barclay W., Fodor E., Reis e Sousa C. (2010). RIG-I detects viral genomic RNA during negative-strand RNA virus infection. Cell.

[bib0680] Resa-Infante P., Jorba N., Coloma R., Ortin J. (2011). The influenza virus RNA synthesis machine: advances in its structure and function. RNA Biol..

[bib0685] Resa-Infante P., Recuero-Checa M.A., Zamarreno N., Llorca O., Ortin J. (2010). Structural and functional characterization of an influenza virus RNA polymerase–genomic RNA complex. J. Virol..

[bib0690] Robertson J.S. (1979). 5′ and 3′ terminal nucleotide sequences of the RNA genome segments of influenza virus. Nucleic Acids Res..

[bib0695] Runge S., Sparrer K.M., Lassig C., Hembach K., Baum A., Garcia-Sastre A., Soding J., Conzelmann K.K., Hopfner K.P. (2014). In vivo ligands of MDA5 and RIG-I in measles virus-infected cells. PLoS Pathog..

[bib0700] Saira K., Lin X., DePasse J.V., Halpin R., Twaddle A., Stockwell T., Angus B., Cozzi-Lepri A., Delfino M., Dugan V., Dwyer D.E., Freiberg M., Horban A., Losso M., Lynfield R., Wentworth D.N., Holmes E.C., Davey R., Wentworth D.E., Ghedin E. (2013). Sequence analysis of in vivo defective interfering-like RNA of influenza A H1N1 pandemic virus. J. Virol..

[bib0705] Saito T., Hirai R., Loo Y.M., Owen D., Johnson C.L., Sinha S.C., Akira S., Fujita T., Gale M. (2007). Regulation of innate antiviral defenses through a shared repressor domain in RIG-I and LGP2. Proc. Natl. Acad. Sci. U. S. A..

[bib0710] Saito T., Owen D.M., Jiang F., Marcotrigiano J., Gale M. (2008). Innate immunity induced by composition-dependent RIG-I recognition of hepatitis C virus RNA. Nature.

[bib0715] Satoh T., Kato H., Kumagai Y., Yoneyama M., Sato S., Matsushita K., Tsujimura T., Fujita T., Akira S., Takeuchi O. (2010). LGP2 is a positive regulator of RIG-I- and MDA5-mediated antiviral responses. Proc. Natl. Acad. Sci. U. S. A..

[bib0720] Satterly N., Tsai P.L., van Deursen J., Nussenzveig D.R., Wang Y., Faria P.A., Levay A., Levy D.E., Fontoura B.M. (2007). Influenza virus targets the mRNA export machinery and the nuclear pore complex. Proc. Natl. Acad. Sci. U. S. A..

[bib0725] Schlee M., Roth A., Hornung V., Hagmann C.A., Wimmenauer V., Barchet W., Coch C., Janke M., Mihailovic A., Wardle G., Juranek S., Kato H., Kawai T., Poeck H., Fitzgerald K.A., Takeuchi O., Akira S., Tuschl T., Latz E., Ludwig J., Hartmann G. (2009). Recognition of 5′ triphosphate by RIG-I helicase requires short blunt double-stranded RNA as contained in panhandle of negative-strand virus. Immunity.

[bib0730] Schmidt A., Schwerd T., Hamm W., Hellmuth J.C., Cui S., Wenzel M., Hoffmann F.S., Michallet M.C., Besch R., Hopfner K.P., Endres S., Rothenfusser S. (2009). 5′-Triphosphate RNA requires base-paired structures to activate antiviral signaling via RIG-I. Proc. Natl. Acad. Sci. U. S. A..

[bib0735] Scott P.D., Meng B., Marriott A.C., Easton A.J., Dimmock N.J. (2011). Defective interfering influenza A virus protects in vivo against disease caused by a heterologous influenza B virus. J. Gen. Virol..

[bib0740] Shapiro G.I., Krug R.M. (1988). Influenza virus RNA replication in vitro: synthesis of viral template RNAs and virion RNAs in the absence of an added primer. J. Virol..

[bib0745] Shaw M.L., Palese P., Knipe D.M., Howley P.M. (2013). Orthomyxoviridae. Fields Virology.

[bib0750] Shingai M., Ebihara T., Begum N.A., Kato A., Honma T., Matsumoto K., Saito H., Ogura H., Matsumoto M., Seya T. (2007). Differential type I IFN-inducing abilities of wild-type versus vaccine strains of measles virus. J. Immunol..

[bib0755] Sommereyns C., Paul S., Staeheli P., Michiels T. (2008). IFN-lambda (IFN-λ) is expressed in a tissue-dependent fashion and primarily acts on epithelial cells in vivo. PLoS Pathog..

[bib0760] Steidle S., Martinez-Sobrido L., Mordstein M., Lienenklaus S., Garcia-Sastre A., Staheli P., Kochs G. (2010). Glycine 184 in nonstructural protein NS1 determines the virulence of influenza A virus strain PR8 without affecting the host interferon response. J. Virol..

[bib0765] Strahle L., Garcin D., Kolakofsky D. (2006). Sendai virus defective-interfering genomes and the activation of interferon-beta. Virology.

[bib0770] Strahle L., Marq J.B., Brini A., Hausmann S., Kolakofsky D., Garcin D. (2007). Activation of the beta interferon promoter by unnatural Sendai virus infection requires RIG-I and is inhibited by viral C proteins. J. Virol..

[bib0775] Sun Q., Sun L., Liu H.H., Chen X., Seth R.B., Forman J., Chen Z.J. (2006). The specific and essential role of MAVS in antiviral innate immune responses. Immunity.

[bib0780] Takahasi K., Yoneyama M., Nishihori T., Hirai R., Kumeta H., Narita R., Gale M., Inagaki F., Fujita T. (2008). Nonself RNA-sensing mechanism of RIG-I helicase and activation of antiviral immune responses. Mol. Cell.

[bib0785] Tan K.S., Olfat F., Phoon M.C., Hsu J.P., Howe J.L., Seet J.E., Chin K.C., Chow V.T. (2012). In vivo and in vitro studies on the antiviral activities of viperin against influenza H1N1 virus infection. J. Gen. Virol..

[bib0790] Tapia K., Kim W.K., Sun Y., Mercado-Lopez X., Dunay E., Wise M., Adu M., Lopez C.B. (2013). Defective viral genomes arising in vivo provide critical danger signals for the triggering of lung antiviral immunity. PLoS Pathog..

[bib0795] Tiley L.S., Hagen M., Matthews J.T., Krystal M. (1994). Sequence-specific binding of the influenza virus RNA polymerase to sequences located at the 5′ ends of the viral RNAs. J. Virol..

[bib0800] Tomescu A.I., Robb N.C., Hengrung N., Fodor E., Kapanidis A.N. (2014). Single-molecule FRET reveals a corkscrew RNA structure for the polymerase-bound influenza virus promoter. Proc. Natl. Acad. Sci. U. S. A..

[bib0805] Turan K., Mibayashi M., Sugiyama K., Saito S., Numajiri A., Nagata K. (2004). Nuclear MxA proteins form a complex with influenza virus NP and inhibit the transcription of the engineered influenza virus genome. Nucleic Acids Res..

[bib0810] Turrell L., Lyall J.W., Tiley L.S., Fodor E., Vreede F.T. (2013). The role and assembly mechanism of nucleoprotein in influenza A virus ribonucleoprotein complexes. Nat. Commun..

[bib0815] Uzri D., Gehrke L. (2009). Nucleotide sequences and modifications that determine RIG-I/RNA binding and signaling activities. J. Virol..

[bib0820] Varga Z.T., Ramos I., Hai R., Schmolke M., Garcia-Sastre A., Fernandez-Sesma A., Palese P. (2011). The influenza virus protein PB1-F2 inhibits the induction of type I interferon at the level of the MAVS adaptor protein. PLoS Pathog..

[bib0825] Von Magnus P. (1951). Propagation of the PR8 strain of influenza A virus in chick embryos. II. The formation of incomplete virus following inoculation of large doses of seed virus. Acta Pathol. Microbiol. Scand..

[bib0830] Von Magnus P. (1951). Propagation of the PR8 strain of influenza A virus in chick embryos. III. Properties of the incomplete virus produced in serial passages of undiluted virus. Acta Pathol. Microbiol. Scand..

[bib0835] Vreede F.T., Chan A.Y., Sharps J., Fodor E. (2010). Mechanisms and functional implications of the degradation of host RNA polymerase II in influenza virus infected cells. Virology.

[bib0840] Vreede F.T., Jung T.E., Brownlee G.G. (2004). Model suggesting that replication of influenza virus is regulated by stabilization of replicative intermediates. J. Virol..

[bib0845] Vreede F.T., Ng A.K., Shaw P.C., Fodor E. (2011). Stabilization of influenza virus replication intermediates is dependent on the RNA-binding but not the homo-oligomerization activity of the viral nucleoprotein. J. Virol..

[bib0850] Wang J., Oberley-Deegan R., Wang S., Nikrad M., Funk C.J., Hartshorn K.L., Mason R.J. (2009). Differentiated human alveolar type II cells secrete antiviral IL-29 (IFN-lambda 1) in response to influenza A infection. J. Immunol..

[bib0855] Wang X., Hinson E.R., Cresswell P. (2007). The interferon-inducible protein viperin inhibits influenza virus release by perturbing lipid rafts. Cell Host Microbe.

[bib0860] Wang X., Li M., Zheng H., Muster T., Palese P., Beg A.A., Garcia-Sastre A. (2000). Influenza A virus NS1 protein prevents activation of NF-kappaB and induction of alpha/beta interferon. J. Virol..

[bib0865] Wang Y., Ludwig J., Schuberth C., Goldeck M., Schlee M., Li H., Juranek S., Sheng G., Micura R., Tuschl T., Hartmann G., Patel D.J. (2010). Structural and functional insights into 5′-ppp RNA pattern recognition by the innate immune receptor RIG-I. Nat. Struct. Mol. Biol..

[bib0870] Weber F., Wagner V., Rasmussen S.B., Hartmann R., Paludan S.R. (2006). Double-stranded RNA is produced by positive-strand RNA viruses and DNA viruses but not in detectable amounts by negative-strand RNA viruses. J. Virol..

[bib0875] Weber M., Gawanbacht A., Habjan M., Rang A., Borner C., Schmidt A.M., Veitinger S., Jacob R., Devignot S., Kochs G., Garcia-Sastre A., Weber F. (2013). Incoming RNA virus nucleocapsids containing a 5′-triphosphorylated genome activate RIG-I and antiviral signaling. Cell Host Microbe.

[bib0880] Wisskirchen C., Ludersdorfer T.H., Muller D.A., Moritz E., Pavlovic J. (2011). The cellular RNA helicase UAP56 is required for prevention of double-stranded RNA formation during influenza A virus infection. J. Virol..

[bib0885] Xiao H., Killip M.J., Staeheli P., Randall R.E., Jackson D. (2013). The human interferon-induced MxA protein inhibits early stages of influenza A virus infection by retaining the incoming viral genome in the cytoplasm. J. Virol..

[bib0890] Ye Q., Krug R.M., Tao Y.J. (2006). The mechanism by which influenza A virus nucleoprotein forms oligomers and binds RNA. Nature.

[bib0895] Yoneyama M., Kikuchi M., Natsukawa T., Shinobu N., Imaizumi T., Miyagishi M., Taira K., Akira S., Fujita T. (2004). The RNA helicase RIG-I has an essential function in double-stranded RNA-induced innate antiviral responses. Nat. Immunol..

[bib0900] York A., Hengrung N., Vreede F.T., Huiskonen J.T., Fodor E. (2013). Isolation and characterization of the positive-sense replicative intermediate of a negative-strand RNA virus. Proc. Natl. Acad. Sci. U. S. A..

[bib0905] Zeng W., Sun L., Jiang X., Chen X., Hou F., Adhikari A., Xu M., Chen Z.J. (2010). Reconstitution of the RIG-I pathway reveals a signaling role of unanchored polyubiquitin chains in innate immunity. Cell.

[bib0910] Zhao M., Zhang J., Phatnani H., Scheu S., Maniatis T. (2012). Stochastic expression of the interferon-beta gene. PLoS Biol..

[bib0915] Zimmermann P., Manz B., Haller O., Schwemmle M., Kochs G. (2011). The viral nucleoprotein determines Mx sensitivity of influenza A viruses. J. Virol..

